# Supplementary material for: A Global Screen for Assembly State Changes of the Mitotic Proteome by SEC-SWATH-MS
Source: Cell Syst. 2020 Feb 26;10(2):133–155.e6. doi: 10.1016/j.cels.2020.01.001 (PMC7042714; doi:10.1016/j.cels.2020.01.001)

P51532 | SMCA4_HUMAN | SMARCA4 BAF190A BRG1 SNF2B SNF2L4
Monomer MW [kDa]: 184.646 Monomer expected elution fraction: 35

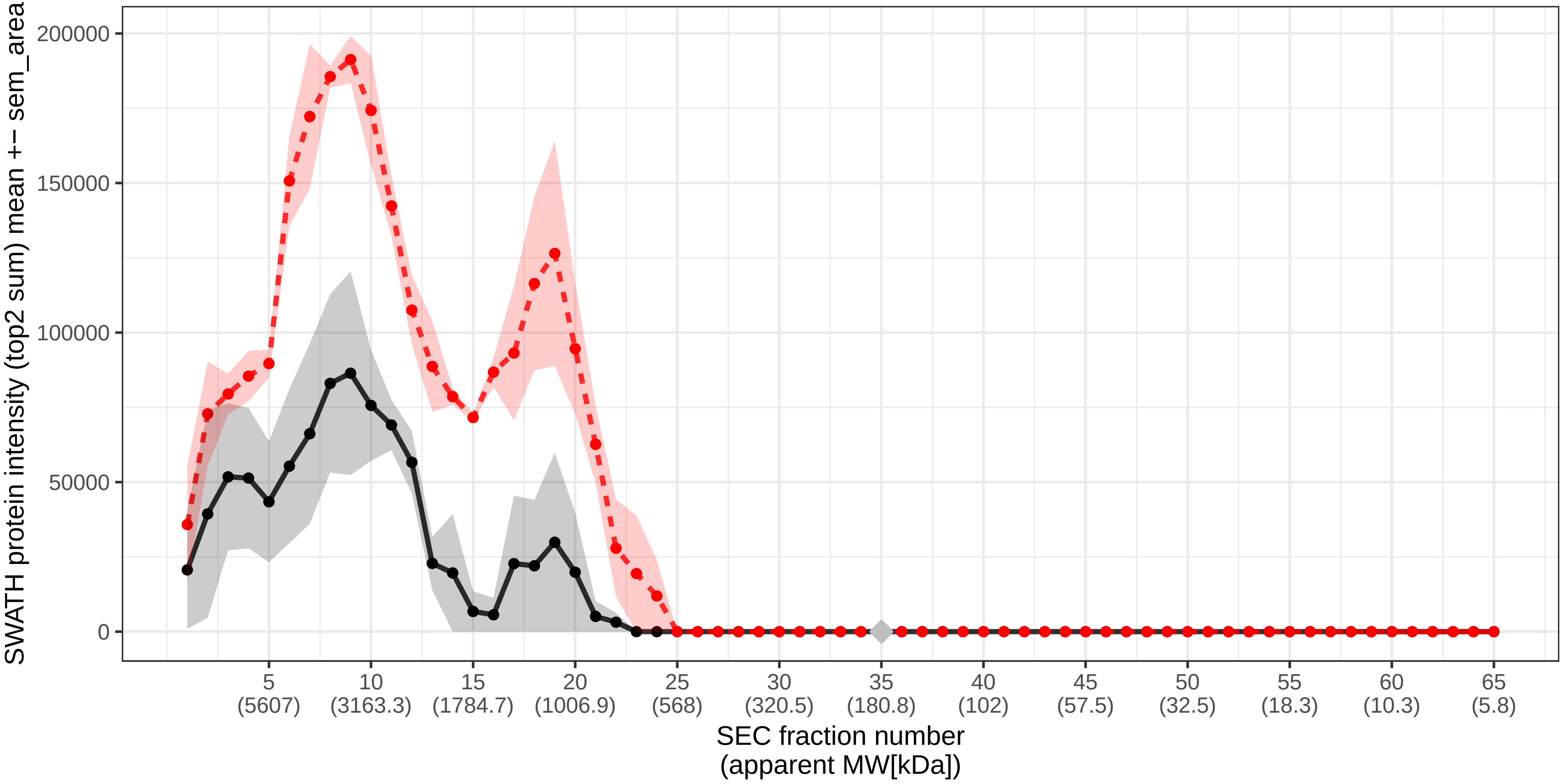

Supplement: Data S1. SEC-SWATH-MS Protein Chromatograms, Related to Figure 1 [file mmc6.zip › SECchrom_P51532_SMCA4_HUMAN_SMARCA4_BAF190A_BRG1_SNF2B_SNF2.pdf]
